# Engineering PRRs: A new path to durable resistance

**DOI:** 10.1016/j.abiote.2026.100028

**Published:** 2026-02-12

**Authors:** Dongjiao Wang, Zhankun Li, Wentao Yang, Youxiong Que, Ming Chang

**Affiliations:** a State Key Laboratory of Tropical Crop Breeding, Institute of Tropical Bioscience and Biotechnology, Sanya Research Institute, Chinese Academy of Tropical Agricultural Sciences, Sanya, 572024, China; b Shijiazhuang Academy of Agriculture and Forestry Sciences, Shijiazhuang, 050041, China; c Key Laboratory of Soybean Disease and Pest Control (Ministry of Agriculture and Rural Affairs), College of Life Sciences, Nanjing Agricultural University, Nanjing, 210095, China

**Keywords:** Broad-spectrum disease resistance, Crop, Immune receptor, nlp20, PRR, RLP23

## Abstract

Engineering the *Arabidopsis* cell-surface immune receptor RECEPTOR-LIKE PROTEIN 23 (RLP23) enhances plant immunity by enabling functional signaling triggered by the conserved pathogen peptide nlp20. Structural optimization enables broad-spectrum disease resistance across species without compromising growth, demonstrating the potential of rationally reprogrammed pattern recognition receptors as a durable and sustainable strategy for crop protection.

## 1 Rethinking resistance breeding through cell-surface immune receptors

**Fig. 1:**
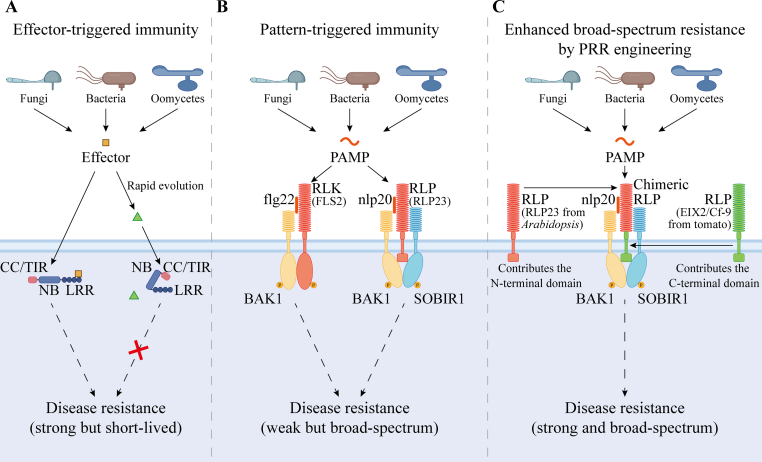
From effector-triggered immunity (ETI) to engineered pattern recognition receptors (PRRs) for durable and broad-spectrum disease resistance. **A** Intracellular nucleotide-binding leucine-rich repeat (NB-LRR, NLR) receptors, typically composed of an N-terminal coiled-coil (CC) or Toll/interleukin-1 receptor (TIR) domain, recognize pathogen effectors to activate ETI. Although ETI confers strong and specific resistance, it is often short-lived because pathogen effectors rapidly evolve to evade recognition. **B** Cell-surface PRRs, including receptor-like kinases (RLKs) and receptor-like proteins (RLPs), detect conserved pathogen-associated molecular patterns (PAMPs) and activate pattern-triggered immunity (PTI). For example, the RLK protein FLAGELLIN-SENSING 2 (FLS2) recognizes bacterial flg22 and signals through the co-receptor BRASSINOSTEROID INSENSITIVE 1-ASSOCIATED KINASE 1 (BAK1), while the RLP23 detects the conserved peptide nlp20 derived from necrosis- and ethylene-inducing peptide-like proteins (NLPs) and signals via the co-receptors BAK1 and SUPPRESSOR OF BIR1-1 (SOBIR1). PTI responses are generally weaker than ETI but provide broad and durable protection. **C** Cross-species engineering of PRRs enables enhanced and stable immune signaling. The *Arabidopsis* RLP23, which recognizes the conserved peptide nlp20 from diverse fungi, oomycetes, and bacteria, was optimized by swapping its C-terminal region with that from the tomato RLP ETHYLENE-INDUCING XYLANASE RECEPTOR 2 (EIX2) or CLADOSPORIUM FULVUM RESISTANCE PROTEIN 9 (Cf-9). These chimeric receptors achieved stronger PTI activation without fitness cost. This schematic illustrates the general concept of PRR engineering, in which the C-terminal domain from one species (Plant A) and the N-terminal domain from another (Plant B) can be combined to produce receptors with improved signal transduction and broad-spectrum disease resistance. Solid lines indicate direct relationships; dashed lines indicate indirect links.

Traditional crop disease-resistance breeding has mainly focused on nucleotide-binding leucine-rich repeat (NB-LRR, NLR) receptors, which detect pathogen effectors and promote effector-triggered immunity (ETI). This strategy provides strong protection, however, pathogen effectors rapidly mutate, causing frequent resistance breakdowns and making NLR-based resistance short-lived in the field (Fig. 1A) [1].

Attention has shifted to cell-surface immune receptors, known as pattern recognition receptors (PRRs), including two main classes: receptor-like kinases (RLKs) and receptor-like proteins (RLPs) [2]. Located at the plasma membrane, RLKs and RLPs recognize conserved pathogen- or damage-associated molecular patterns (PAMPs/DAMPs) and activate pattern-triggered immunity (PTI) [2]. PTI responses are generally milder than ETI but tend to be broader and more durable, making PRRs attractive targets for engineering long-term, wide-spectrum resistance (Fig. 1B).

Interestingly, cross-species transfer of PRRs often results in limited functionality since receptor and co-receptor interactions and downstream signaling components such as SUPPRESSOR OF BIR1-1 (SOBIR1) and BRASSINOSTEROID INSENSITIVE 1-ASSOCIATED KINASE 1 (BAK1) differ among plant species [3]. The recent study by Yang et al. overturns this trend by demonstrating that the *Arabidopsis* RLP23 protein can be engineered and transferred into diverse crops, where functional immune signaling is restored or amplified through C-terminal domain replacement, enhancing resistance without penalty to growth [4]. This signals a shift from purely NLR-based breeding to a network-based design of immune receptors.

## 2 Structure-guided reengineering of RLP23 enhances immune signaling

The *Arabidopsis* RLP23 protein recognizes nlp20, a highly conserved 20-amino acid peptide derived from necrosis- and ethylene-inducing peptide-like proteins (NLPs) that are widely present in fungi, oomycetes, and bacteria [5]. Perception of nlp20 triggers typical PTI responses such as accumulation of ethylene and reactive oxygen species (ROS) and activation of defense-related genes, providing a broad and stable layer of resistance (Fig. 1B) [5].

Structural analysis revealed that the C-terminal region of RLP23, consisting of juxtamembrane (JM), transmembrane (TM), and intracellular (IC) domains, is essential for signal transduction mediated by the co-receptors SOBIR1 and BAK1 [4]. In *Nicotiana benthamiana*, deletion of the IC domain weakened nlp20-induced ethylene responses, whereas deletion of the full C-terminal region caused an even stronger reduction, indicating that C-terminal domains are essential for receptor activation in heterologous systems [4].

Replacing the IC or C-terminal domains of RLP23 with those from tomato RLPs ETHYLENE-INDUCING XYLANASE RECEPTOR 2 (EIX2) and CLADOSPORIUM FULVUM RESISTANCE PROTEIN 9 (Cf-9) greatly enhanced immune signaling in *N. benthamiana* without changing nlp20 recognition [4]. Domain swaps using RLPs from tomato, rice, or poplar further amplified responses, suggesting that species-specific C-terminal features optimize the compatibility between receptors and co-receptors [4]. These findings establish the C-terminal region of RLP23 as a tunable module controlling signaling output and enable functional cross-species transfer of PRRs through downstream signaling adaptation (Fig. 1C).

## 3 Engineered RLP23 confers broad resistance without fitness cost

Introducing the engineered RLP23 variants into *Arabidopsis* and *N. benthamiana* markedly enhanced resistance to multiple necrotrophic and hemibiotrophic pathogens producing NLP effectors [4]. Plants expressing domain-swapped receptors exhibited stronger ethylene accumulation, elevated ROS bursts, and up-regulation of defense marker genes, demonstrating robust PTI [4]. Importantly, these modifications did not impose observable penalties on growth or reproduction, indicating that receptor activation remained precisely regulated [4].

The absence of fitness costs is a crucial achievement. Many constitutively active or overexpressed immune receptors cause dwarfism or yield loss due to chronic defense activation [6]. In contrast, the reengineered RLP23 variants retained low basal activity while mounting rapid and strong responses only upon ligand recognition, highlighting the advantage of signaling-competent but conditionally activated receptor design [4].

Moreover, the engineered receptors could maintain compatibility with endogenous co-receptors SOBIR1 and BAK1, ensuring efficient signal transduction [4]. The combination of enhanced responsiveness, functional stability, and growth neutrality highlights the feasibility of designing precision-tuned PRRs for sustainable disease resistance across crops (Fig. 1C).

## 4 Paving the way for sustainable and resilient crop immunity

The reengineering of RLP23 exemplifies a rational approach to strengthening PTI through structural optimization of PRRs. This work is part of a growing movement toward precision immune design, which aims to improve disease resistance while maintaining plant vigor and yield.

A compelling precedent is translational regulation of defense genes using upstream open reading frames (uORFs). Traditional transcriptional overexpression of immune regulators, such as the salicylic acid receptor *NONEXPRESSER OF PATHOGENESIS-RELATED GENES 1* (*NPR1*) in *Arabidopsis*, often leads to constitutive defense and growth penalties [7]. A *TL1-BINDING FACTOR 1* (*TBF1*)-derived uORF cassette that allows NPR1 translation only upon pathogen attack, conferring robust resistance in rice without yield loss [8]. This innovation demonstrates how post-transcriptional control can reconcile immunity with productivity, providing a regulatory “buffer” for engineered defense activation.

At the intracellular immune receptor level, interfamily co-transfer of *NLR* pairs offers another paradigm. Transfer of the pepper *Bs2* sensor and its helper *NLR REQUIRED FOR CELL DEATH* (*NRC*) into rice reconstitutes effector recognition against *Xanthomonas oryzae* pv. *oryzicola*, generating resistant lines with unaltered field performance [9]. The success of this cross-family receptor reconstruction reveals the feasibility of transplanting complete immune modules across taxonomic boundaries.

More recently, researchers systematically mined over 13,000 LRR-RLKs to identify SELECTIVE COLD SHOCK PROTEIN RECEPTOR (SCORE), a PRR detecting bacterial cold-shock protein peptides [10]. Guided by structural and phylogenomic analyses, synthetic SCORE variants were engineered to recognize diverse pathogens, including *Ralstonia*, *Xanthomonas*, and *Candidatus Liberibacter asiaticus*, demonstrating a scalable framework for receptor discovery and functional reprogramming [10].

Together, these advances mark a turning point from gene-by-gene improvement to network-level engineering of plant immunity. By integrating structure-based PRR reconfiguration, translational fine-tuning, and modular receptor transfer, future breeding can produce ideal crops with durable, broad-spectrum, and fitness-neutral resistance, thereby guiding a new era of resilient agriculture.

## CRediT authorship contribution statement

**Dongjiao Wang:** Writing – original draft. **Zhankun Li:** Writing – original draft. **Wentao Yang:** Writing – original draft. **Youxiong Que:** Writing – review & editing. **Ming Chang:** Writing – review & editing.

## Declaration of competing interest

The authors declare no competing interests.

## Data Availability

No data was used for the research described in the article.
